# The Role of Iron in Intestinal Mucus: Perspectives from Both the Host and Gut Microbiota

**DOI:** 10.1016/j.advnut.2024.100307

**Published:** 2024-09-26

**Authors:** Shuan Liu, Jie Yin, Dan Wan, Yulong Yin

**Affiliations:** 1 Laboratory of Animal Nutritional Physiology and Metabolic Process, Key Laboratory of Agro-ecological Processes in Subtropical Region, Institute of Subtropical Agriculture, Chinese Academy of Sciences, Changsha, Hunan, China; 2 University of Chinese Academy of Sciences, Beijing, China; 3 College of Animal Science and Technology, Hunan Agriculture University, Changsha, China

**Keywords:** iron, mucus, goblet cells, gut microbiota, intestinal inflammation

## Abstract

Although research on the role of iron in host immunity has a history spanning decades, it is only relatively recently that attention has been directed toward the biological effects of iron on the intestinal mucus layer, prompted by an evolving understanding of the role of this material in immune defense. The mucus layer, secreted by intestinal goblet cells, covers the intestinal epithelium, and given its unique location, interactions between the host and gut microbiota, as well as among constituent microbiota, occur frequently within the mucus layer. Iron, as an essential nutrient for the vast majority of life forms, regulates immune responses from both the host and microbial perspectives. In this review, we summarize the iron metabolism of both the host and gut microbiota and describe how iron contributes to intestinal mucosal homeostasis via the intestinal mucus layer with respect to both host and constituent gut microbiota. The findings described herein offer a new perspective on iron-mediated intestinal mucosal barrier function.

**Statement of Significance**

This review is the first to systematically discuss the regulation of iron on the intestinal mucus barrier from the perspective of the host and gut microbiota.

## Introduction

Iron is an essential micronutrient involved in a range of cellular functions, including DNA repair, oxygen transport, energy production, and mitochondrial function [1,2], and consequently, deficiencies in this metal can have diverse physiological effects ranging from simple anemia to complex pathologies. For example, iron deficiency can exacerbate cardiovascular disease by modulating the ejection fraction [3], promotes liver cancer metastasis by downregulating the angiogenesis-associated glutamyl aminopeptidase [4], and induces enteritis by casing an increase in intestinal permeability [5]. Conversely, iron overload can contribute to inducing oxidative stress and ferroptosis, leading to the onset of a range of disorders, including heart and liver failure, as well as intestinal inflammation and even gastric cancer [[6], [7], [8]]. In microorganisms, iron serves as a cofactor for proteins involved in essential microbial metabolic pathways, including redox reactions, DNA synthesis, and the production of short-chain fatty acids (SCFAs) [9], and accordingly, the proliferation and growth of almost all microbiota, including both commensal and pathogenic species, are dependent on the utilization of unabsorbed dietary iron. Iron deficiency has been found to be associated with reductions in the abundance and diversity of commensal microbiota, compromising colonization and resistance and increasing the likelihood of pathogenic microbial colonization and invasion [10]. However, certain pathogenic bacteria, such as those in the genera *Helicobacter*, *Desulfovibrio*, and *Shigella*, have evolved tolerance mechanisms to elevated iron concentrations, and thus by promoting the proliferation of these bacteria, an excess of dietary iron can exacerbate intestinal inflammation [[11], [12], [13], [14]]. Consequently, both a deficiency and surfeit of iron can be detrimental to the host.

The intestinal mucosal barrier is the first line of defense against pathogenic and nonpathogenic microorganisms. A key component of this barrier is the mucus layer composed of a gel-forming mucin secreted by goblet cells, of which mucin2 (MUC2) is the main functional component [[15], [16], [17]]. This material covers the intestinal epithelium, serving as a direct barrier to pathogen invasion [[18], [19], [20], [21], [22]]. The function of the mucus layer is influenced by goblet cell differentiation and function (mucin synthesis, maturation, and secretion), as well as the destruction of the mucus layer by pathogenic bacteria. The presence of this mucus covering is particularly important in the colon, given that Paneth cells, which have been established to secrete antimicrobial peptides, such as lysozyme and defensins, are absent in the colons of rodents and are also notably rare in human descending colon [[23], [24], [25]]. Recently, the mucus layer in the distal colon has been found to comprise 3 layers, namely, the niche (encapsulating the contents), a thick layer, and a thin layer [23], among which the thin layer has been established to comprise sulfated *O*-glycosylated mucus from the distal colon, whereas the other layers are formed by *O*-glycosylated mucus secreted by the proximal colon [23]. However, most studies have focused on the thin or thick mucus layer, often referred to as the outer or inner mucus layer, respectively [24,25]. Although the outer mucus layer serves as a habitat for commensal bacteria, such as *Bacteroides thetaiotaomicron*, *Akkermansia muciniphila*, and *Escherichia coli*, the inner mucus layer impermeably separates these commensal bacteria from the epithelial cells [[26], [27], [28], [29]]. Thus, the close juxtaposition of these discrete mucus layers makes them key sites for host–microbiota interactions.

The pathogenesis of intestinal inflammation includes alterations in host genetics and gut microbiology, both of which can influence mucus layer function [30], and the findings of several studies have revealed that damage to the mucus layer, resulting from host- or microbially induced alterations can contribute to intestinal inflammation. For example, it has been shown that *NLRP6*^*−/−*^ goblet cells are unable to synthesize mucus proteins, which can thus disrupt the integrity of the intestinal mucus layer, thereby reducing host resistance to pathogenic bacteria [25]. Similarly, *Vamp8*^*−/−*^ mice have been found to be highly susceptible to infection due to a thinner mucus layer and an increase in encounters with microbial antigens [31]. From a microbiological perspective, it has been shown that bacteria in the genus *Olsenella* can inhibit the colonization of vancomycin-resistant *Enterococcus*, a multidrug-resistant bacteria that threatens public health, in the intestinal tract through nutrient consumption from mucus [32]. Additionally, it has been found that whereas germ-free mice lack colonic mucus production [33], mucus secretion was effectively restored in conventionalized littermates in which the intestinal flora was structurally similar [23].

Similarly, patients with colitis have been shown to have a thinner colonic mucus layer [34], whereas a reduction in the main component of mucus, MUC2, in patients with active ulcerative colitis has been found to be correlated with the diminished secretory activity of goblet cells in response to microbial assault [35]. Furthermore, it has been demonstrated that the concentration of phosphatidylcholine in the mucus of patients with colitis is lower than that in healthy individuals [36] and that by restoring mucus barrier function, treatment with exogenous phosphatidylcholine can effectively alleviate the inflammation in patients [37]. Collectively, these clinical findings highlight the significance of the mucus layer in preventing intestinal inflammation.

In this article, we review the associations among iron, the mucus layer, and intestinal inflammation from both host and gut microbiological perspectives, with a particular focus on ascertaining how iron regulates host responses and the gut microbiota, and in turn, how these host responses and gut microbiota influence the intestinal mucus layer.

## Host Iron Metabolism

**FIGURE 1:**
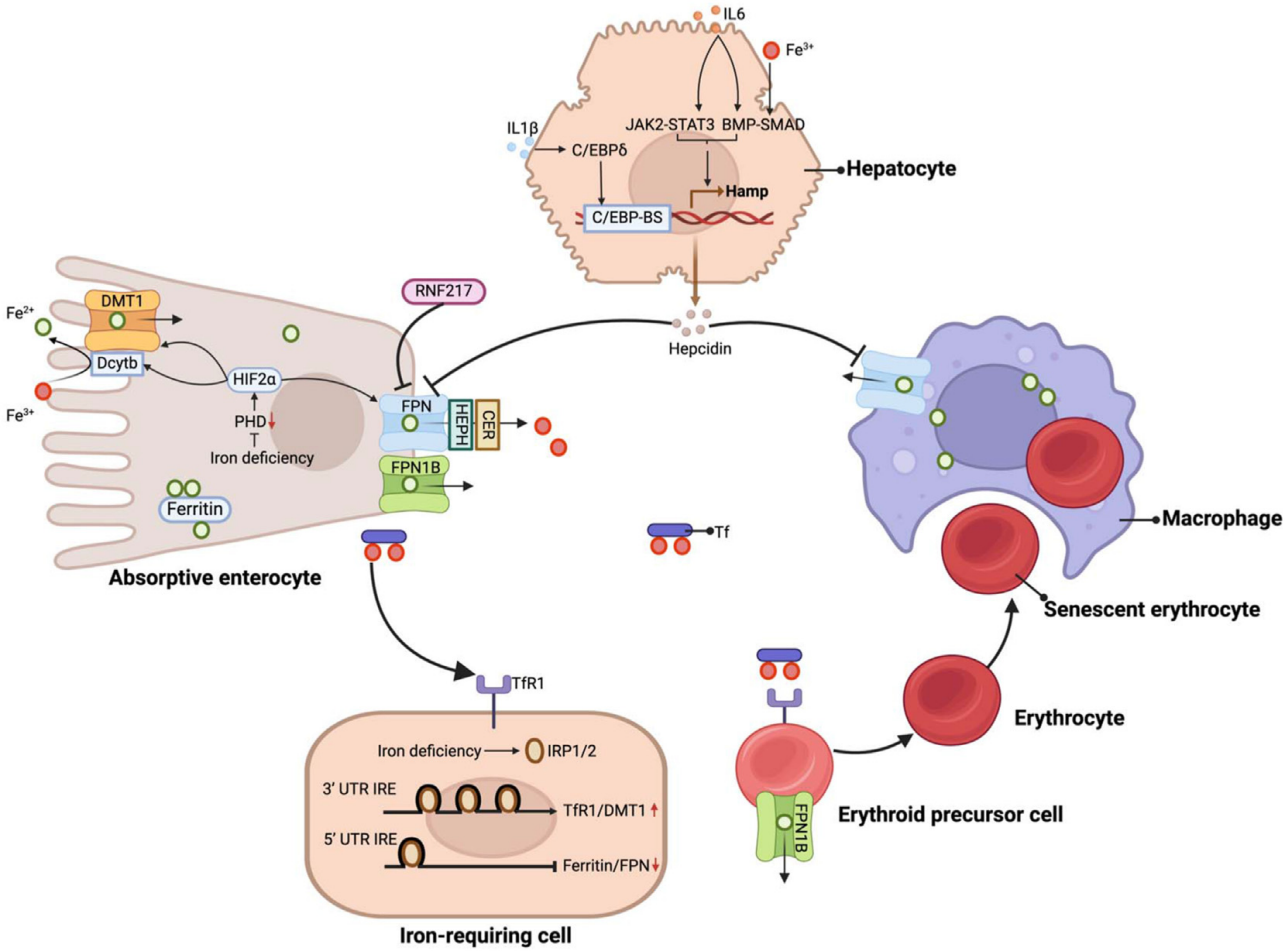
Iron metabolism. Nonheme iron is reduced to ferrous ions by Dcytb and subsequently enters enterocytes via DMT1. Cells other than enterocytes obtain iron mainly through the activity of TfR1. The ferrous ions in cells are primarily stored by ferritin and used in a range of metabolic process or transported by FPN. The degradation of FPN is regulated by hepcidin and RNF217. By sensing a low-iron status in enterocytes, PHD stabilizes the expression of HIF-2α, which promotes the expression of Dcytb, DMT1, and FPN to enhance the absorption and utilization of iron. Under conditions of iron deficiency, IRP1/2 binds to the 3ʹ UTR of IRE on TfR1 and DMT1, promoting their expression. Simultaneously, IRP1/2 binds to the 5ʹ UTR of IRE on ferritin and FPN, thereby inhibiting their expression. In iron deficiency, the expression of IRP1/2 is activated. However, duodenal epithelial cells and erythroid precursor cells can circumvent IRP-mediated repression of FPN under iron-deficient conditions by expressing FPN1B, which lacks the 5ʹ IRE. Iron ions circulating within the blood are derived not only from the absorption of iron by epithelial cells in the intestinal lumen but also from the phagocytosis of senescent erythrocytes by macrophages. Hepcidin, partially regulated by cytokines and iron, is derived mainly from hepatocytes. Figure created with BioRender.com. BMP, bone morphogenetic protein; BS, binding site; C/EBP, CCAAT enhancer-binding protein; CER, ceruloplasmin; Dcytb, duodenal cytochrome B reductase; DMT1, divalent cation transporter 1; FPN, ferroportin; HEPH, hephaestin; HIF, hypoxia-inducible factor; IRE, iron-responsive element; IRP, iron regulatory protein; JAK, Janus kinase; PHD, prolyl hydroxylase domain; RNF217, ring finger protein 217; SMAD, mothers against decapentaplegic homolog; STAT, signal transducer and activator of transcription; Tf, transferrin; TfR, transferrin receptor; UTR, untranslated region.

The absorption and metabolism of iron within the intestines have been summarized in previous studies [[38], [39], [40]] and are shown in Figure 1. Generally, iron is absorbed in the form of heme and nonheme iron in the duodenum. The uptake of heme iron by intestinal epithelial cells (IECs) is assumed to be mediated by the proton-coupled folate transporter and hemoxygenase-1, whereas nonheme iron is reduced to Fe^2+^ by duodenal cytochrome B reductase (Dcytb) at the tip of the villi within the intestinal epithelium and subsequently enters the intestinal epithelium via divalent cation transporter 1 (DMT1) [41,42]. Absorbed iron atoms are present in intestinal cells as either free iron or in combination with ferritin, and in response to systemic iron demand, free iron or iron dissociated from ferritin via nuclear receptor coactivator 4 is excreted through ferroportin (FPN), the main iron exporter [43,44]. Ferrous iron is immediately oxidized by ceruloplasmin circulating in the plasma and hephaestin anchored to enterocytes and thereafter binds to transferrin (Tf) to form Tf-Fe^3+^ in the blood [[45], [46], [47], [48], [49]], which further binds to transferrin receptor 1 (TfR1) to supply iron for erythrocyte synthesis or tissue metabolism [50]. Macrophages recycle senescent erythrocytes for iron reuse, with this recycled iron constituting the main source of iron available to the host [51]. The key regulator of systemic iron is hepcidin, mainly secreted by the liver, which binds to FPN and promotes its degradation, thereby suppressing the intracellular iron efflux pathway and contributing to a reduction in systemic iron content [[52], [53], [54]]. Notably, Jiang et al. [55] have recently found that ring finger protein 217 (RNF217), an E3 ubiquitin ligase, also promotes FPN degradation.

The role of iron status in hepcidin regulation has been well reported [56], with elevated levels of iron being found to promote an upregulation of hepcidin expression. However, the regulatory role played by hypoxia-inducible factor (HIF) 2 alpha in the local iron metabolism of intestinal cells should not be overlooked. HIF-2α, which promotes the expression of DMT1, Dcytb, and FPN, is regulated by iron-dependent prolyl hydroxylase domain (PHD) enzymes that sense the intracellular iron concentration. Low intracellular iron concentrations cause a reduction in PHD enzyme activity, thereby activating HIF-2α expression, which consequently promotes iron absorption and excretion via DMT1, Dcytb, and FPN [57]. Recent findings have indicated that low levels of hepcidin expression in the liver lead to high FPN expression in intestinal cells, followed by an increase in the excretion of intracellular free iron, which in turn reduces PHD enzyme activity and subsequently activates HIF-2α expression, promoting an increase in iron uptake [58]. Additionally, intracellular iron concentrations have been established to be regulated by the iron regulatory protein/iron-responsive element (IRP/IRE) system. In iron-deficient states, in response to the mRNA expression of iron uptake-related proteins DMT1 and TfR1, either IRP1 or IRP2 is activated by binding to the 3ʹ UTR of the corresponding IRE, whereas binding to the 5ʹ UTR results in inhibition of the expression of ferritin and FPN. It is noteworthy that in duodenal epithelial cells and erythroid precursor cells, FPN1B is expressed, which lacks the 5ʹ IRE and can evade IRP-dependent repression under iron-deficient conditions. This mechanism ensures effective iron absorption and the restriction of erythropoiesis in the context of systemic iron deficiency [59].

Notably, the metabolism of iron is also regulated by inflammatory factors, with hepcidin acting as a bridge. For example, IL6 is known to activate hepcidin transcription via the Janus kinase (JAK) 2 signal transducer and activator of transcription (STAT) 3 pathway or the bone morphogenetic protein (BMP)-suppressor of mothers against decapentaplegic (SMAD) pathway [[60], [61], [62]]. However, inflammation-induced IL1β can activate hepcidin transcription by promoting the expression of the CCAAT enhancer-binding protein δ, which binds to the putative CCAAT enhancer-binding protein-binding site within the hepcidin promoter [63].

## The Role of Iron in the Intestinal Microbiota

**FIGURE 2:**
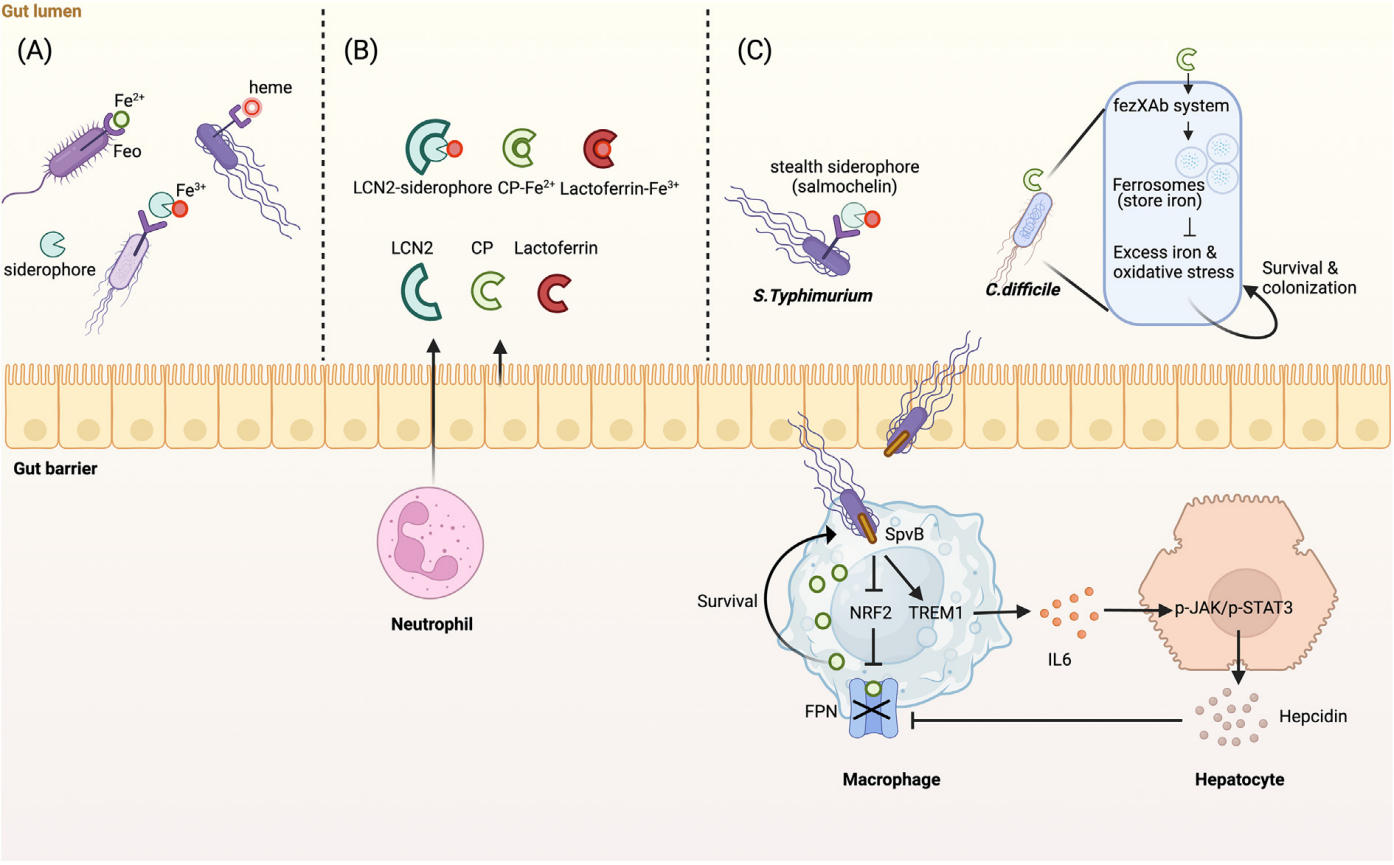
The role of iron in intestinal microbes. (A) Gut bacteria acquire iron through Feo, siderophores, or binding to heme. (B) Although the host can block bacterial access to iron by producing LCN2, CP, and lactoferrin, certain bacteria survive by adopting novel strategies for acquiring iron. (C) *Clostridioides difficile* uses LCN2 to produce ferrosomes as a means of storing iron, thereby mitigating the damage caused by iron overload and iron deficiency. Similarly, *Salmonella typhimurium* gains access to iron by producing salmochelin that contributes to the evasion LCN2 interception. In addition, *S. typhimurium* ensures the adequacy of its iron sources via intracellular and systemic coregulation of FPN following host invasion. Figure created with BioRender.com. CP, calprotectin; FPN, ferroportin; JAK, Janus kinase; LCN2, lipocalin-2; NRF2, nuclear factor erythroid-derived 2-related factor 2; SpvB, Salmonella plasmid virulence B; STAT, signal transducer and activator of transcription; TREM1, triggering receptor expressed on myeloid cells 1.

Almost all bacteria require iron for survival and metabolism, and from the perspective of host health, microbial iron uptake has been linked to the virulence of most human pathogens, whereas sequestration of iron from bacteria is considered an efficient strategy for host defense [64]. The mechanisms associated with iron uptake by iron-demanding microbiota and host sequestration are summarized in Figure 2.

Numerous studies have reported on the influence of iron on intestinal microbial communities. An excess of iron can promote increases in the abundance of pathogenic bacteria, including *Salmonella typhimurium* and *Clostridioides difficile* [65,66]. Conversely, elevated iron levels are associated with reductions in the abundance of probiotic strains, such as those in the genera *Lactobacillus* and *Bifidobacterium* [[67], [68], [69], [70], [71]]. Notably, by suppressing HIF-2α expression, 1,3-diaminopropane and reuterin produced by *Lactobacillus* contribute to a reduction in host intestinal iron absorption [72], which may explain why, compared with other flora, *Lactobacillus* species have a greater growth advantage under low-iron conditions. Furthermore, the availability of iron often enhances the proliferation of and virulence factor expression in pathogenic bacteria, thereby facilitating the invasion of mucus and enhancing infection [73]. Additionally, iron has been shown to promote increases in the abundance of neutral bacteria, including *Ruminococcus gnavus* [74,75].

Gut microorganisms typically have efficient mechanisms for acquiring iron (Figure 2A), including binding to organic molecules such as heme [76], the Feo system (a widely distributed transport system of Fe^2+^ in bacteria) [77], and the production of siderophores (secondary metabolites of bacteria with strong iron affinity) that form soluble Fe^3+^complexes in the environment, which are subsequently absorbed by specific bacterial receptors [78]. However, the host can prevent iron uptake by pathogenic bacteria by releasing lactoferrin (chelating Fe^3+^), lipocalin-2 (LCN2) (sequestering siderophores), and calprotectin (CP) (sequestering Fe^2+^) from neutrophils or IECs at the sites of infection [[79], [80], [81]] (Figure 2B). However, the LCN2-based defense response appears to be ineffective against *S. typhimurium*, which produces certain stealth siderophores, such as salmochelin, that contribute to an evasion of the released LCN2 [82]. Moreover, *Salmonella* plasmid virulence B (SpvB), an important virulence effector protein in *Salmonella*, has been shown to dysregulate host iron metabolism to ensure sufficient iron for survival and virulence. Specifically, SpvB reduces FPN expression by inhibiting nuclear factor erythroid-derived 2-related factor 2 (NRF2), thereby reducing intracellular iron efflux [83]. Systematically, SpvB ensures the maintenance of intracellular iron concentrations by activating triggering receptor expressed on myeloid cells 1 (TREM1), an inflammatory amplifier that promotes IL6 production, subsequently activating HAMP transcription via the JAK-STAT3 pathway, which in turn promotes the degradation of FPN [84] (Figure 2C). Additionally, elevated levels of host CP activate the *C. difficile* intracellular ferrosome (fezXAB) system to form membrane-bound ferrosome organelles containing amorphous iron phosphate. These ferrosomes store iron, effectively mitigating the harmful effects of iron overload and oxidative stress on *C. difficile*, thereby enhancing its survival and colonization [85] (Figure 2C). However, despite the effective iron acquisition strategies adopted by pathogenic bacteria to enhance their survival and virulence, the intact mucus layer generally serves as an effective barrier isolating these microbes from the host [86,87].

## Regulation of Intestinal Microbiota-Mediated Iron in Mucus Layers

**FIGURE 3:**
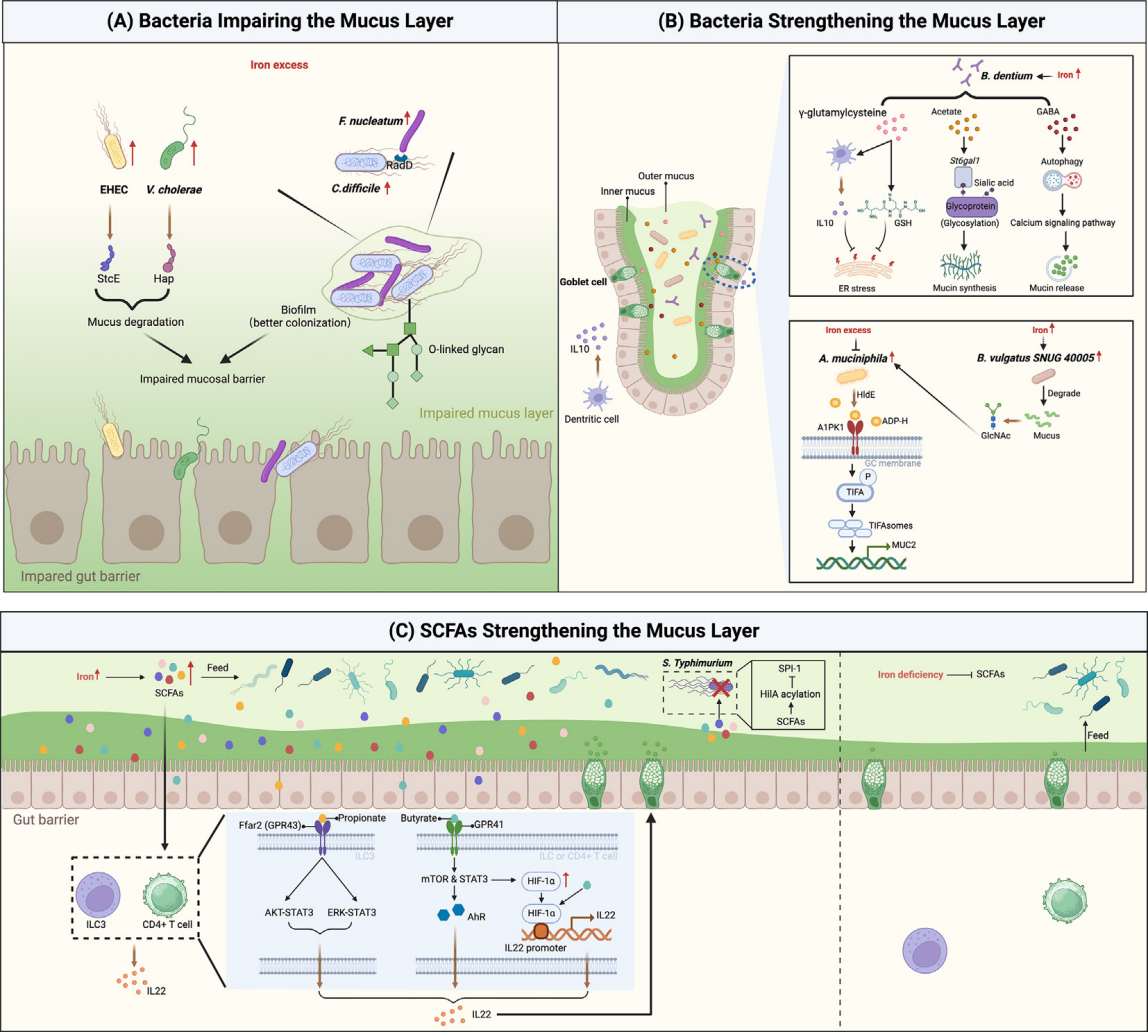
The effects of gut microbiota and metabolites on the mucus layer. (A) Excess iron promotes the growth of pathogenic bacteria. Enzymes produced by these pathogenic bacteria degrade the mucus layer. Additionally, interactions among certain pathogenic bacteria can facilitate the formation of biofilms that are more effective in colonizing the mucus layer. Collectively, these strategies contribute to the pathogenicity of these bacteria. (B) Moderate iron supplementation enhances the production of γ-glutamylcysteine, acetate, and GABA, and improves goblet cell function to promote MUC2 production by upregulating the abundance of *Bifidobacterium dentium*. Additionally, it may promote increases in the abundance of *Akkermansia muciniphila*, thereby promoting MUC2 production by upregulating the abundance of *Bacteroides vulgatus SNUG 40005*. However, when present in excess, iron inhibits increases in the abundance of *A. muciniphila.* (C) Moderate iron supplementation enhances the production of SCFAs, among which, propionate and butyrate promote the production of IL22 by stimulating ILC3 or CD4+ T cells, thereby enhancing mucus barrier function. Additionally, SCFAs contribute to inhibiting the virulence factors of *S. typhimurium*. Iron deficiency, however, suppresses the production of SCFAs, thereby accelerating consumption of the mucus layer by Mucinophilus. Figure created with BioRender.com. A1PK1, alpha kinase 1; ADP-H, ADP-heptose; AhR, aryl hydrocarbon receptor ; AKT, protein kinase B; EHEC, enterohaemorrhagic *E. coli*; ER, endoplasmic reticulum; ERK, extracellular signal-regulated kinase; GABA, γ-aminobutyric acid; GlcNAc, N-acetylglucosamine; GPR, G-protein coupled receptor; GSH, glutathione; HIF, hypoxia-inducible factor; ILC3, group 3 innate lymphoid cell; mTOR, mammalian target of rapamycin; MUC2, mucin 2; SCFA, short-chain fatty acid; SPI-1, Salmonella pathogenicity island-1; St6gal1, ST6 *N*-acetylgalactosaminide α-2,6-sialyltransferase; STAT, signal transducer and activator of transcription; StcE, secreted protease the of C1-esterase inhibitor; TIFA, TNF receptor associated factor-interacting protein with forkhead-associated domain.

In response to the influence of iron, pathogenic bacteria can penetrate or disrupt the mucus layer, thereby exerting their respective pathogenic effects. Conversely, commensal bacteria colonizing the outer mucus layer act on the intestinal mucus layer to protect the host from intrusion and play a pivotal role in maintaining intestinal homeostasis. The roles of iron-demanding microbes in the mucus layer are summarized in Figure 3.

### Pathogenic bacteria

The activity of pathogenic bacteria, including enterohaemorrhagic *E. coli* (EHEC), *Vibrio cholerae*, *C. difficile*, and *Fusobacterium nucleatum*, can lead to an imbalance in the gut microbiota and contribute to disease development. The key step in infection is the primary contact between bacterial and host epithelial cells; however, enteropathogenic bacteria must initially pass through (disrupt) the mucus layer, a process facilitated by deployment of the flagellum/flagella [88]. In addition, a number of pathogens destabilize host tissue by secreting enzymes that cleave mucus proteins (Figure 3A).

The availability of iron is essential for supporting the pathogenesis of EHEC, which secreted protease of C1-esterase inhibitor (StcE), a metalloprotease that can attach to and degrade mucins, thereby facilitating the passage of pathogens to epithelial cells and the subsequent elicitation of disease [89]. Similarly, *V. cholerae*, a human pathogen that causes a severe diarrheal disease associated with devastating epidemics, has a definite requirement for iron and secretes a soluble Zn^2+^-dependent metalloproteinase (haemagglutinin protease) that has mucinolytic activity [90,91].

By facilitating colonization of the mucus layer, intermicrobial interactions can also promote pathogenesis (Figure 3A). In this context, it has been established that iron stimulates the growth of *C. difficile* in a dose-dependent manner, and the production of toxin A, a factor necessary for the pathogenesis of *C. difficile,* is elevated in response to iron treatment [92]. In *F. nucleatum*, iron necessary for survival is acquired via a process involving the lysis and disruption of erythrocytes mediated by its DNA hunger/stationary phase protective proteins [93]. Recently, it has been shown that *C. difficile* attaches to MUC2 by binding to *O*-linked glycans, whereas *F. nucleatum* promotes robust biofilm formation by binding its RadD protein to the flagella of *C. difficile*. Notably, the formation of such biofilms facilitates mucus layer colonization by both *F. nucleatum* and *C. difficile* [94], infection by which can contribute to the progression of colorectal cancer as well as cause diarrhea [95,96].

### Commensal bacteria

Commensal bacteria coexist with the host in nonpathogenic associations, and their presence can contribute to inhibiting the colonization of pathogenic bacteria [97]. In this regard, it has been shown that the severely damaged mucus layer of aseptic mice could be effectively restored by transplanting fecal bacteria from normal mice, thereby indicating the essential roles played by commensal bacteria in mucus secretion [23]. Figure 3B summarizes the mechanisms by which certain iron-demanding commensal bacteria make a positive contribution to the production of mucus.

Whereas the findings of a number of studies have indicated that an increase in iron has the effect of reducing the abundance of *Bifidobacterium* [71,98], lower abundances of *Bifidobacterium dentium* have been detected under conditions of anemia [99]. *B. dentium* has been established to have a limited ability to degrade mucus due to a lack of mucin-associated glycosyl hydrolases [100]. Additionally, this human-derived species colonizes the outer mucus layer, wherein certain metabolites produced by this bacterium have been found to contribute to enhancing goblet cell function. For example, γ-glutamylcysteine has been shown to promote glutathione (GSH) production by epithelial cells, or IL-10 derived from dendritic cells, which inhibit endoplasmic reticulum (ER) stress in goblet cells, as indicated by reductions in the production of reactive oxygen species (ROS) and expression of glucose-regulated protein 78, C/EBP homologous protein (CHOP), and X-box binding protein 1 (XBP1) [101]. In addition, acetate promotes sialic acid glycosylation via the expression of ST6 *N*-acetylgalactosaminide α-2,6-sialyltransferase (St6gal1), thereby facilitating mucin synthesis, whereas γ-aminobutyric acid (GABA) stimulates the calcium signaling pathway via autophagy, thus promoting the release of mucus granules. By enhancing mucus layer function, *B. dentium* can contribute to protecting against colitis.

Although species of *Bacteroides* have been reported to have mucin-degrading activity, recent studies have shown that *N*-acetylglucosamine produced by the human isolate *Bacteroides vulgatus SNUG 40005* during mucus degradation promotes growth of the commensal bacterium *Akkermansia muciniphila* as well as restoration of the mucus layer [102]. Although the effects of iron on *B. vulgatus SNUG 40005* have yet to be reported, the findings of one study indicated that the abundance of *B. vulgatus* is enhanced in response to iron supplementation [103]. ADP-heptose, a microbiota-associated molecular pattern produced by *A. muciniphila* via the HldE enzyme, is recognized and bound by alpha kinase 1, a newly identified pattern recognition receptor that is highly expressed in goblet cells. The ensuing phosphorylation of TNF receptor associated factor-interacting protein with forkhead-associated domain (TIFA) induces the formation of large TIFA oligomers, referred to as TIFAsomes, that can promote the expression of MUC2, thereby contributing to the integrity of the intestinal mucosal barrier [104]. However, although the abundance of *A. muciniphila* can be promoted in response to an elevation in iron levels mediated by an increase in the abundance of *B. vulgatus SNUG 40005*, it has also been shown that the abundance of *A. muciniphila* is reduced under conditions of iron overload [65].

*R. gnavus*, the abundance of which increases in response to iron supplementation, can promote the expression of MUC2 in colonic goblet cells [105], probably by producing SCFAs (see below for details). Additionally, the findings of numerous studies have shown that populations of the main species positively associated with mucus barrier function, including those in the family *Muribaculaceae*, increase in response to iron supplementation. Conversely, the abundances of species, such as those in the genus *Lactobacillus*, that are negatively associated with mucus function, increase under conditions of iron deficiency [33,74,[106], [107], [108], [109]]. However, it has yet to be established whether these bacteria affect MUC2 or whether MUC2 influences the abundance of these bacteria.

The aforementioned findings thus indicate that excess iron levels contribute to the destruction of the mucus layer by pathogenic bacteria and prevent beneficial bacteria from reinforcing this barrier, thereby increasing the susceptibility to inflammation. However, iron supplementation (in moderation) can contribute to reinforcing the mucus layer against infection by certain commensal bacteria.

### Metabolites

Among the multitude of microbial metabolites released into the gut, SCFAs have emerged as key factors involved in immune regulation [[110], [111], [112]]. Figure 3C illustrates the roles of SCFAs in the mucus layer in response to iron supplementation. Several studies have shown that iron supplementation contributes to increases in the concentration of SCFAs, including acetate, butyrate, propionate, valerate, and isovalerate [[113], [114], [115]], mainly by promoting the abundance of SCFA-associated microbiota, such as those in the genus *Clostridium* and family *Ruminococcaceae* [[116], [117], [118]]. A recent study found that SCFAs contribute to restoring the intestinal mucosal barrier, including increasing the number of MUC2-producing goblet cells, mediated via the G-protein coupled receptor (GPR) 41/43 signaling pathway, thereby alleviating inflammatory bowel disease (IBD) [119]. Consistently, it has been shown that SCFAs, especially propionate, can activate Ffar2 (GPR43), a metabolite-sensing receptor that promotes intracellular IL22 production by group 3 innate lymphoid cells (ILC3s) via activation of the protein kinase B (AKT)-STAT3 or extracellular signal-related kinase (ERK)-STAT3 axes. By maintaining mucosal barrier integrity, including mucin production, this activation subsequently reduces susceptibility to enteritis [120]. Additionally, the binding of butyrate to GPR41 promotes IL22 production by CD4+ T and ILC cells, thereby inhibiting colitis. The specific mechanisms involve an upregulation of the aryl hydrocarbon receptor by the activated transcription factors STAT3 and mTOR, which in turn promotes IL22 production. Alternatively, activated STAT3 can promote IL22 production by upregulating HIF-1α, which subsequently binds to the IL22 promoter, an interaction that is facilitated by butyric acid via histone modification [121]. However, an excess of butyrate has been found to impair the proliferation of intestinal stem cells and hampers epithelial repair [122].

In addition to strengthening the intestinal mucosal barrier by activating GPRs to regulate signaling pathways, SCFAs act directly on pathogenic bacterial virulence factors and in doing so reduce the invasion of IECs and transmission in vivo. For example, butyric acid inhibits *Salmonella* pathogenicity island 1 (*SPI-1*) gene expression by acylating HilA, a key transcriptional regulator of *SPI-1* gene expression in *S. typhimurium*, thereby attenuating disruption of the mucosal barrier and subsequent invasion [123].

However, SCFAs also serve as a primary source of energy for intestinal microorganisms [124], and the glycoproteins in the mucus layer can also be used as a source of nutrients for certain colonic microorganisms (Mucinophilus), including commensal bacteria and invading enteric pathogens. Under conditions in which the levels of SCFAs are reduced, the microbiota become more dependent on endogenous nutrients, including mucins, to sustain their metabolism. Thus, the mucus layer can be damaged to varying extents, and it has been demonstrated that in such circumstances, mice become increasingly susceptible to invasion by pathogenic bacteria [124]. These findings accordingly support the hypothesis that the endogenous niche created by host glycans is more detrimental in the absence of SCFAs.

## Regulation of Host-Mediated Iron in Mucus Layers

**FIGURE 4:**
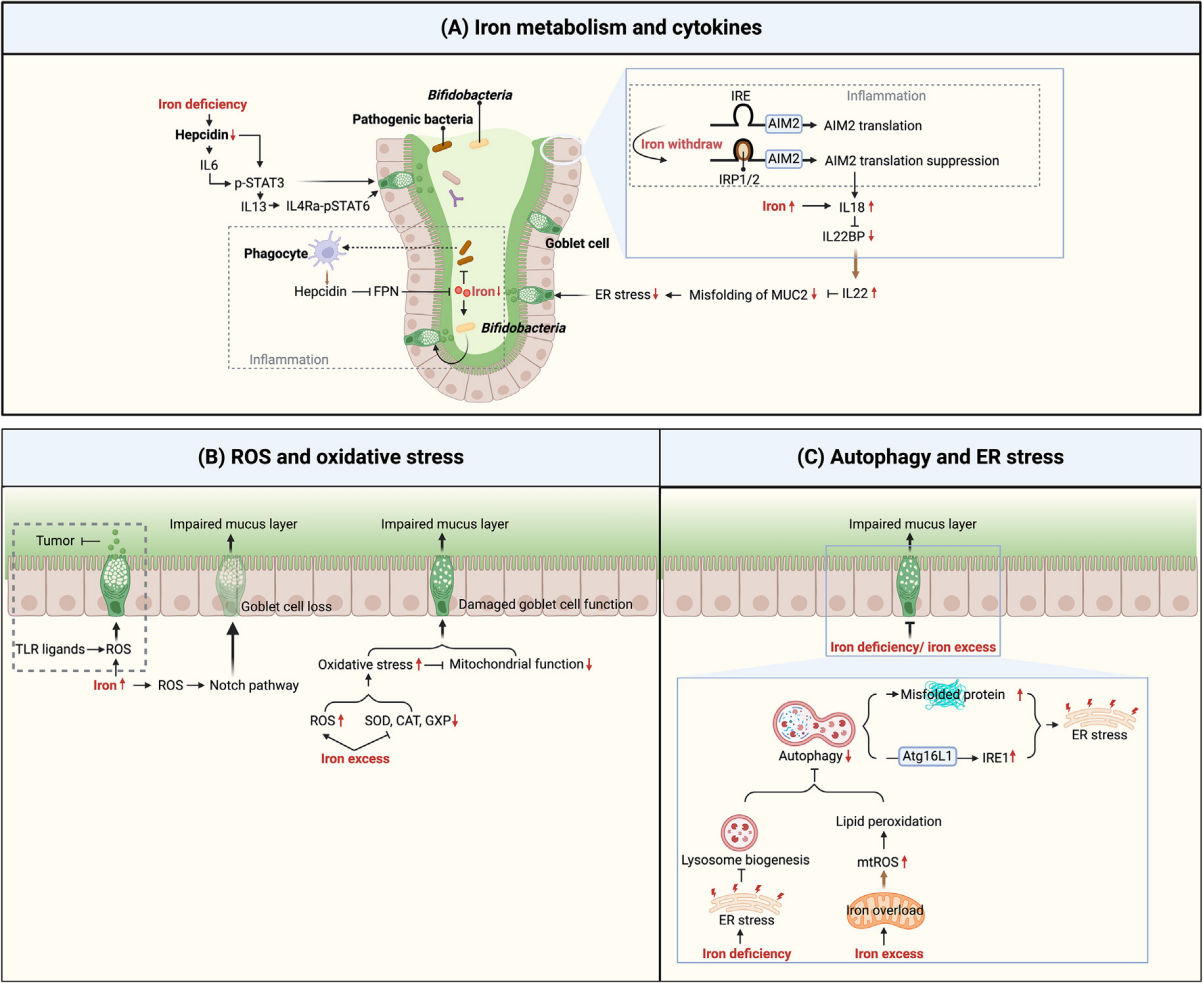
The effects of host iron levels and metabolism on the mucus layer. (A) Systemic downregulation of hepcidin due to iron deficiency may have a positive effect on mucosal barrier function by promoting the upregulation of IL6 and IL13. However, the upregulation of local hepcidin expression due to inflammation may enhance intestinal mucosal barrier function via microbial interactions. Sufficient iron under normal conditions and low-iron levels associated with inflammation could protect the integrity of the intestinal barrier by promoting the production of IL22. (B and C) Both iron deficiency and excess impair the mucus layer by inducing ER or oxidative stress. Figure created with BioRender.com. AIM2, absent in melanoma 2; BP, binding protein; CAT, catalase; ER, endoplasmic reticulum; FPN, ferroportin; GXP, glutathione peroxidase; IRE, iron-responsive element; IRP, iron regulatory protein; mtROS, mitochondrial reactive oxygen species; MUC2, mucin 2; ROS, reactive oxygen species; SOD, superoxide dismutase; STAT, signal transducer and activator of transcription; TLR, toll-like receptor.

Iron has a direct effect on goblet cell function in a manner that is independent of the gut microbiota [74], although the precise underlying mechanisms have yet to be reported. In this regard, altered cytokine secretion associated with iron metabolism is a key feature of intestinal inflammation and plays a pivotal role in intestinal mucosal immunity [125,126]. In addition, intestinal goblet cells have been shown to be highly sensitive to different stresses. Figure 4 summarizes the effects of immune-related cytokines and different stress responses associated with iron metabolism that are closely related to goblet cell function in the intestinal mucosa.

### Cytokines

Cytokines are secreted or membrane-presented molecules that mediate developmental and immune functions, the significance of which is increasingly being recognized. Indeed, cytokine/cytokine receptor engineering is being effectively applied in disease therapy, including the treatment of autoimmune disorders, cancer, and viral infections [127]. In this section, we provide an overview of the cytokines that are regulated by iron metabolism and involved in the regulation of goblet cell function.

As previously mentioned in the “iron metabolism” section, hepcidin is regulated by inflammatory factors; however, it is also worth noting that inflammatory factors are in turn regulated by hepcidin (Figure 4A). Iron deficiency-induced reduction in hepcidin promotes the expression of IL6 and STAT3 [128]. IL6 promotes mucus secretion via STAT3 signaling in IECs to enhance barrier function [11], and IL6 levels are invariably positively correlated with MUC2 [129]. It is presumed that a deficiency in hepcidin due to iron deficiency, increasing IL6, promotes mucosal immune function by acting directly on epithelial cells, including goblet cells. Additionally, hepcidin deficiency due to iron deficiency is associated with a promotion of IL13 secretion by macrophages via the STAT3 pathway in the heart [130]. By acting via the IL13-IL4Ra-STAT6 axis, IL13 promotes goblet cell proliferation in the type II immune response, thereby enhancing mucus secretion and amphiregulin expression, and thus contributing to the repair and protection of mucosal barrier function [131]. However, in dextran sodium sulfate (DSS)-induced inflammation (with bleeding), it has been shown that the expression of hepcidin in type 2 conventional dendritic cells in the lamina propria of the colon is maintained, possibly due to the induction of bacteria and bacteria-derived metabolites. By acting on FPN-expressing phagocytes, hepcidin prevents the efflux of iron from phagocytes into the blood, thereby limiting the availability of iron for pathogenic bacteria in the intestinal lumen and promoting the abundance of *Bifidobacteria*, thereby enhancing mucosal barrier function [132]. This is consistent with the earlier conclusion that, in inflammatory situations, the host triggers an iron isolation mechanism that prevents pathogenic bacteria from using iron to support infective proliferation. Hence, the systemic downregulation of hepcidin contributes to enhancing the integrity of mucosal barrier by inducing the upregulation of IL6 and IL13. Conversely, the activation of hepcidin expression in response to an excess of iron is well established. However, under inflammatory conditions, the upregulation of local hepcidin expression via bacteria may also contribute to enhancing the intestinal mucosal barrier.

IL22 and IL18 function as inflammatory factors and thereby provide a link between iron nutrition and intestinal inflammation (Figure 4A). IL22 has also been established to alleviate ER stress by correcting the misfolding of MUC2 protein in goblet cells, thereby maintaining the integrity of the mucosal barrier [133,134], whereas immune and epithelial IL18 have been demonstrated to inhibit goblet cell maturation by regulating the transcriptional program governing goblet cell development [135]. However, enteric nervous system-derived IL18 has been shown to be essential for host protection against *Salmonella* infection, mediated via goblet cell antimicrobial peptide (*Ang4*, *Retnlb*, and *Itln1*) production rather than goblet cell differentiation (*Klf4*) or mucus production (*Muc2*) [136]. Iron has been shown to promote the expression of IL18 in macrophages [137]. Additionally, in DSS-induced inflammation, deficiency of the inflammasome sensor absent in melanoma 2 (AIM2) induces IL18 production, consequently inhibiting IL22 binding protein expression and promoting an increase in IL22 production [138]. Surprisingly, a newly discovered isoform of AIM2, predominantly expressed during inflammation, has a longer 5ʹ UTR containing an IRE. Robinson et al. [139] have also shown that higher levels of iron can lead to an inactivation of IRP1/2, which binds to the 5ʹ UTR of IRE (as described above), thereby inhibiting the translation of AIM2. These findings thus provide evidence to indicate that low levels iron of during inflammation and iron sufficiency under normal conditions could contribute to protecting the integrity of the mucus barrier via IL22 production.

Iron invariably promotes the upregulated expression of IL1β [137], TNFα [140], IL4 [141], and IL10 [142], with the levels of IL1β and TNFα consistently showing a trend opposite to that of MUC2 expression [[143], [144], [145]] whereas the levels of IL4 and IL10 consistently mirror the trend in MUC2 expression; however, the underlying mechanisms have yet to be reported.

### Stress responses

Both oxidative and ER stress are involved in the regulation of goblet cell function, and the regulatory effects of iron on these 2 stress types are extensively documented. In this section, we review the influence of iron on the mucus layer mediated via its modulation of oxidative and ER stress.

Both ROS and the oxidative stress induced by excessive ROS accumulation can adversely influence development of the mucus layer (Figure 4B). Iron can promote the generation of ROS via the Fenton reaction or the mitochondrial electron transport chain [146,147]. A recent study showed that under conditions of stimulation with toll-like receptor ligands, an increase in ROS levels enhances MUC2 exocytosis, thereby contributing to prevention of colonic tumor development [148]. However, in a further study, elevated ROS levels exacerbated pathogen invasion by activating the Notch pathway and regulating intestinal secretory cell differentiation, thereby resulting in the loss of goblet cells and damage to the mucus layer [149]. Excess iron induces oxidative stress by promoting ROS accumulation or by reducing the activity of key antioxidant enzymes, such as superoxide dismutase, catalase, and GSH peroxidase, subsequently impairing mitochondrial function, as evidenced by a reduction in ATP production [150]. Oxidative stress and impaired mitochondrial function contribute to a disruption of the secretory functions of intestinal goblet cells [151]. These findings indicate that iron acts on goblet cells via ROS with variable effects. However, oxidative stress caused by iron overload can impair goblet cell function and the integrity of the mucus layer.

ER stress is a key bridge that mediates the role of iron in mucus layer formation (Figure 4C). By inducing ER stress, iron deficiency leads to a reduction in autophagy [associated with an upregulated expression of p62 and microtubule-associated protein 1A/1B-light chain 3 B (LC3B) II], as indicated by phospho-PERK and CHOP, subsequently disrupting protein folding and maturation associated with lysosome biogenesis, indicated by the downregulation of lysosome-associated membrane protein 1 [152]. By inducing mitochondrial oxidative stress, a deficiency in iron activates the IRE-XBP1-CHOP signaling pathway in the ER, leading to ER stress [153]. Conversely, an iron overload can contribute to an inhibition of autophagy, although independent of ER stress mediation. Instead, excess iron tends to accumulate in the mitochondria, inducing the overproduction of mitochondrial ROS, which leads to lipid peroxidation and ultimately to impaired autophagic flux [152]. By reducing misfolded proteins in goblet cells or by removing IRE1a in an Atg16L1-dependent manner [154], autophagy reduces ER stress, thereby stimulating an overproduction of mucus, resulting in a thicker and denser mucus layer that effectively protects the host from colitis. However, in goblet cells, the development of ER stress is governed by an intrinsic switch that controls mucus secretion, it is also dependent on microbes and nucleotide-binding oligomerization domain-containing protein 2 (NOD2) [155]. Generally, both iron deficiency and excess can contribute to disrupting goblet cell function and mucus layer development by inhibiting autophagy and inducing ER stress.

## The Effects of Intestinal Inflammation and Impaired Mucus Layers on Iron Metabolism

**FIGURE 5:**
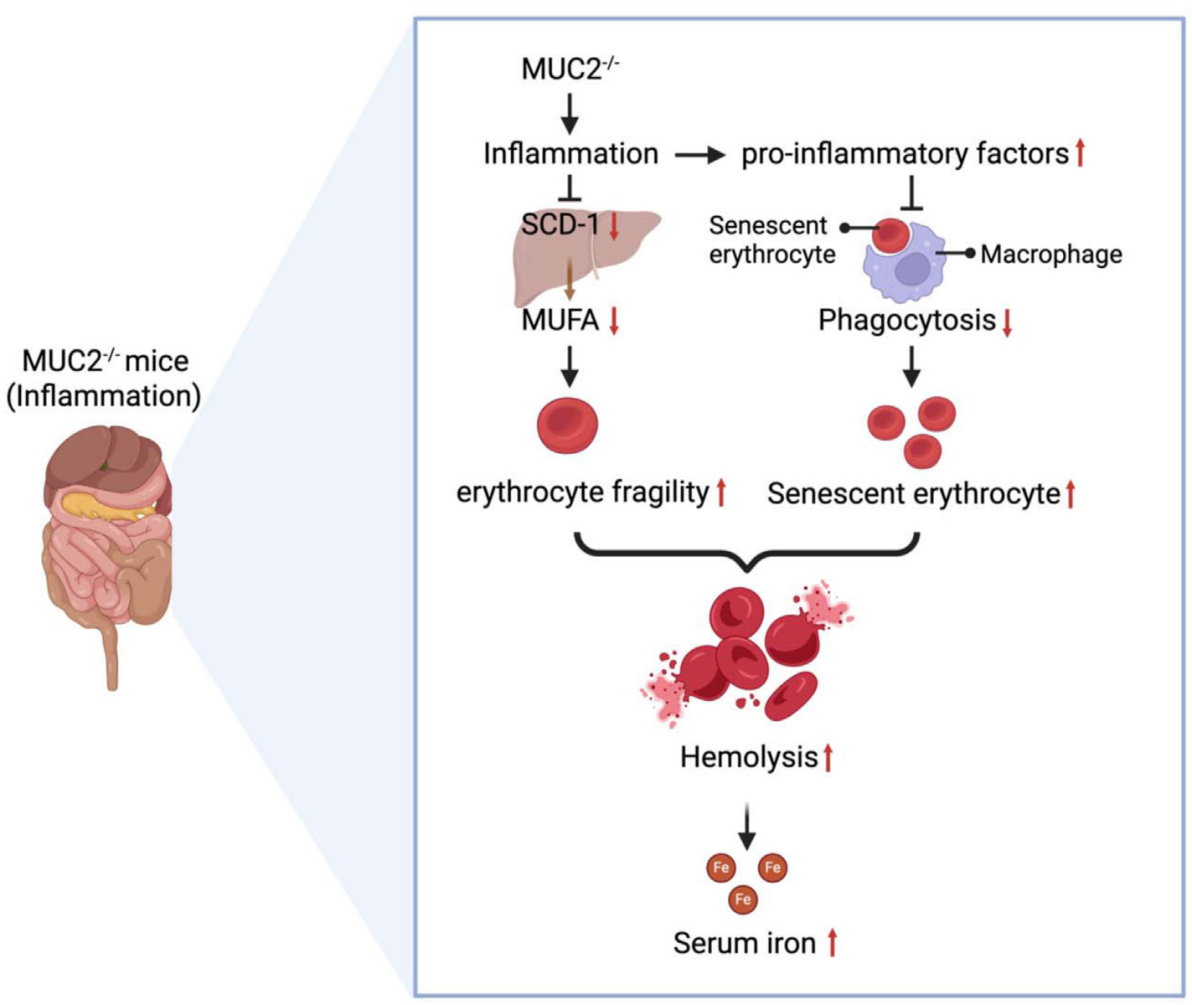
The effects of mucin2 on iron metabolism. *Muc2*^*−/−*^*-*induced inflammation in mice leads to an increase in hemolysis and elevated levels of serum iron, mediated by increases in erythrocyte membrane fragility and attenuation of the ability of macrophages to phagocytose senescent erythrocytes. This occurs independent of genotype. Figure created with BioRender.com. MUC2, mucin 2; MUFA, monounsaturated fatty acid; SCD, stearoyl-CoA desaturase.

As described in the previous section, both iron deficiency and overload can lead to intestinal inflammation via a disruption of the mucus barrier. Figure 5 shows how iron is regulated in response to the onset of intestinal inflammation induced by an impaired mucus layer. In this regard, it has been shown that *Muc2*^*−/−*^ mice are characterized by increased intestinal mucosal barrier permeability, as a consequence of mucus layer loss and impaired functioning of tight junction proteins [156], which can be accompanied by IBD due to the underlying inflammation [157]. Recently, it has been demonstrated that *Muc2*^*−/−*^ mice have elevated serum levels of total iron, as a consequence of heightened intestinal permeability and basal inflammation rather than their genotype [158]. Specifically, it is believed that underlying inflammation contributes to an increase in erythrocyte fragility by downregulating stearoyl coenzyme A desaturase-1 in the liver, thereby contributing to a reduction in the production of monounsaturated fatty acids, which are considered key components in maintaining the fluidity of the erythrocyte membrane. In addition, proinflammatory factors impair the ability of spleen-derived macrophages to phagocytose senescent erythrocytes. Both these mechanisms result in the delayed removal of circulating erythrocytes, hemolysis, and an increase in serum total iron content, thereby exacerbating bacterial infections, which can eventually lead to the development of sepsis.

However, this scenario tends to contradict the traditional concept that IBD leads to the development of iron deficiency anemia (IDA) [159], in which elevated levels of IL6 in IBD patients activate hepcidin expression in the liver. Iron efflux from macrophages and small IECs are the primary sources of circulating iron, and the FPN produced by both these cell types is degraded by hepcidin, the pathway for iron efflux from cells, resulting in a deficiency in circulating iron and leading to IDA [160]. Under inflammatory conditions, this reduction in circulating iron levels appears to have a protective effect on the host by limiting the availability of iron for pathogenic bacteria, thereby inhibiting their growth. This could be because the severity of IBD induced by a substantial reduction in the mucus layer due to a loss of MUC2 is higher than that of conventional IBD, thereby highlighting the importance of the mucosal layer in preventing the onset of IBD.

## Perspectives

In this review, we examine the effects of iron on the mucus layer from the perspectives of both the gut microbiota and mammalian hosts, and in doing so, provide new insights into the role of iron in modulating intestinal mucosal immunity and regulating intestinal inflammation. However, it is still necessary to gain a more precise and in-depth understanding at the host level. For example, although it has been established that iron can directly ameliorate MUC2 production and mitigate the invasion of *S. typhimurium* in a gut microbiota-independent manner, the underlying genetic mechanisms have not been completely elucidated. Additionally, with respect to the aforementioned studies that have examined the effects of cytokines or different stress responses on the mucus layer, although potential microbial involvement was not mentioned, there were no specific data to indicate a definite absence of microbial involvement. Consequently, potential involvement of the gut microbiota in the observed effects should not be overlooked. Furthermore, evidence indicates that ER stress in goblet cells (which control mucin secretion) is dependent on NOD2. Accordingly, further research using host-only models (such as germ-free animals) is needed to refine our understanding of the regulatory roles played by iron in determining the mucus layer function from a host perspective.

Dysbiosis of the gut microbiota, disruption of cytokine secretion, and oxidative/ER stress, induced either by iron overload or deficiency, have been identified as key factors contributing to the regulation of intestinal inflammation via the mucus layer, which accordingly explains the intestinal inflammation (characterized by poor absorption and residual excess iron in the intestinal lumen) associated with iron deficiency or oral iron supplementation in clinical settings. Additionally, an integration of clinical phenotypes and animal experimental data has indicated that by enhancing the integrity of the mucus layer, administering novel iron supplements (with lower doses and better absorption) prior to the onset of inflammation may contribute to reinforcing intestinal mucosal barrier function.

## Author contributions

The authors’ responsibilities were as follows – SL: drafting and revising the manuscript; JY, DW, YY: contributed to outlining the article and provided suggestions for revisions; and all authors: read and approved the final version.

## Funding

This work was supported by the National Natural Science Foundation of China (32372827), the Science and Technology Innovation Program of Hunan Province (2023RC3201), the Youth Innovation Promotion Association of Chinese Academy of Sciences (2022370), the Postdoctoral Fellowship Program of China Postdoctoral Science Foundation (GZB20230830), the China Agriculture Research System of MOF and MARA, and the National Center of Technology Innovation for Pigs.

## Conflict of interest

The authors report no conflicts of interest.
